# Fertility and age

**DOI:** 10.4103/0974-1208.74152

**Published:** 2010

**Authors:** Korula George, Mohan S Kamath

**Affiliations:** Reproductive Medicine Unit, Christian Medical College Hospital, Vellore, Tamil Nadu, India

**Keywords:** Female age, FSH, ovarian reserve

## Abstract

The changing social scenario together with economic growth and an increase in job opportunities has to a great extent reduced gender inequality and has resulted in more and more older women seeking help from infertility clinics. Fertility and aging have always been closely linked and the age of the female partner remains the single most important factor in predicting success with treatment. Although tests for the ovarian reserve are an important informative tool and are helpful in selecting treatment options, they are poor predictors of the outcome.

## INTRODUCTION

Infertility is a distressing issue to both the couple and their families. Recognizing its importance, the World Health Organization has classified it as a global health problem.[1] The ability to procreate is age related, more so in the female partner. In the female fetus, germ cell proliferation comes to a standstill by around 20 weeks, resulting in women being born with a set number of primordial follicles. In contrast, active sperm production in men continues throughout the adult life, with age causing mainly a decline in function. At birth, the female child is born with about 5 million primordial follicles which decrease to about 500,000 at menarche. With each subsequent menstrual cycle follicular atresia/apoptosis continues with the numbers decreasing to about 25,000 at the age of 37 and 1000 close to menopause.[2] Naturally, there is an age-related decline in fecundity, the decrease usually starting at the age of 32 with a dramatic fall after the age of 37. Spelt differently, the natural monthly fecundity rate which is about 25% between 20 and 30 years of age decreases to below 10% above the age of 35.[3]

The number of older women approaching fertility clinics for treatment in India is increasing. The intentional delay in child bearing, facilitated by the availability of effective contraception, can be attributed to several factors. Increasing educational/job opportunities for members of both the sexes result in an ambitious pursuit of careers, often resulting in late marriages. Although the double income no kids concept helps to achieve financial stability and satisfy materialistic wants, eventually the inherent desire for children surfaces. The significance of female age with regard to fertility is still ill-understood, even among the educated.

Increasing female age is also associated with both obstetric and gynecological problems. The increased incidence of spontaneous abortions most often due to aneuploidy together with common obstetrics complications like preeclampsia, preterm labor, and intrauterine growth retardation are common.[4]

Since spermatogenesis is a continuous process, the focus on the effects of paternal age has received limited attention. The likelihood for conception, spontaneous or otherwise, in older women with partners above the age of 40 is reduced, and pregnancies are associated with a higher incidence of complications like miscarriages, congenital anomalies, and genetic disorders.[5,6] Down’s syndrome has also been linked to increasing paternal age.[6] These poor outcomes are attributed to sperm DNA damage.

A diminished ovarian reserve can be either physiological (age related) or due to a premature decline in the reserve. The clinician needs to be attuned to the effect of female age on fertility outcomes and render appropriate advice with early referral to an infertility specialist when required. Although infertility is defined as an inability to conceive following a year of unprotected intercourse, this definition needs to be modified in the older woman. Investigation and treatment should be initiated earlier in women 35 and above. The assessment of the ovarian reserve helps in counselling as well as determining appropriate therapy. There is also a clear distinction between physiological and premature diminished ovarian reserves with the latter having a better prognosis in terms of clinical pregnancies.

Establishing a woman’s ovarian reserve is important as the patient would want to know the probability of success with treatment. Several tests have been advocated. We will briefly catalog the commonly used ones and comment on the newer methods.

## AGE

As mentioned earlier, age is perhaps the single most important factor in assessing an ovarian reserve and reflects both the quantity and quality of oocytes. Not surprisingly, in IVF cycles, older women tend to produce lesser number of oocytes and embryos derived from them have lower implantation potential.[3] Further reflecting on the oocyte quality, women who conceive, experience higher miscarriage rates and increased incidence of congenital anomalies.[3,7]

## SERUM FSH

In women with a diminished ovarian reserve, through the feedback mechanism, low inhibin B levels (associated with a reduced granulosa cell mass) result in elevated serum FSH levels in the early follicular phase.[8] In addition, a decline in luteal phase inhibin A also contributes to elevated FSH levels during luteal–follicular phase transition. FSH levels above 20 mIU/ml are related to poor outcomes.[9] In regularly menstruating women, the intercycle variability of FSH levels is not uncommon. Nevertheless, a single high value should be considered significant.[10] Although elevated basal FSH levels predict a low oocyte yield, they are a poor predictor of IVF success. Thus, serum FSH levels are useful in counselling couples prior to IVF, but should not be used to deny IVF treatment.[11]

## BASAL ESTRADIOL LEVELS: E2 LEVELS

Elevated estradiol levels on day 2/3 of the menstrual cycle, attributed to rapid premature follicle recruitment and consequent loss of pituitary inhibition in women with a poor ovarian reserve, are linked to poor outcomes.[10] Basal estradiol levels have a low predictive value for a poor response and pregnancy.[12,13]

## ANTRAL FOLLICLE COUNT

Using transvaginal ultrasound on day 2/3 of the cycle, it is possible to estimate the number of antral follicles (<10 mm size) present in the ovaries. An antral follicle count (AFC) of 4 or less was associated with higher cancellation and poorer pregnancy rates.[14] AFC may be used as a screening test for possible poor responders but not as a diagnostic test to exclude patients from IVF treatment.[13] The suppressive effect of oral contraceptive pills on the AFC needs to be kept in mind.

## ANTI-MULLERIAN HORMONE

Anti-Mullerian hormone (AMH) is produced by the granulosa cells of the antral and preantral follicles. Serum AMH levels reflect the ovarian pool of primordial follicles. AMH levels remain stable during adulthood decreasing towards menopause. The inter and intracycle variability of AMH is low enough to allow measurement at any time of the cycle. More importantly, AMH levels are not affected by contraceptive pills or GnRH agonists.[15]

Due to these inherent advantages, in day-to-day clinical practice, AMH is gaining popularity as a test for ovarian reserve. Although its role has not been established, AMH together with AFC are considered the better predictors of the ovarian reserve.

Although other tests like inhibin B, clomiphene challenge test, and GnRH agonist stimulation tests have been described, they are not commonly used in clinical practice. Perhaps the most revealing of all tests is the actual response to gonadotrophins. Normal ovarian reserve tests are of no real value when a poor response to an adequate dose of gonadotrophins has been observed. This highlights the fact that none of these tests can be used for diagnostic purposes. They help mainly in counselling patients regarding the probability of a response.

Suspicion of a poor ovarian reserve is usually treated by offering IVF. This is essentially done to prevent the delay in initiating treatment and to obtain oocytes while they are still retrievable. The natural cycle IVF/minimal stimulation protocol has also been advocated in these situations.[16]

## CONCLUSION

Age is a very important factor with regard to fertility, and even with all the advancements in assisted reproduction, it still remains an insurmountable barrier. Public awareness of this fact is important as the age-related decline in fecundity leaves the clinician and the couple with limited treatment options. From a purely fertility aspect, delay in child bearing should be avoided. Tests for predicting the ovarian reserve are useful prognostic tools but poor predictors of the IVF outcome.
